# Genomic exploration and *in silico* prioritization of putative COX-2-targeting metabolites from *Streptomyces* sp. VITGV156 (MCC 4965)

**DOI:** 10.3389/fbinf.2026.1840680

**Published:** 2026-08-18

**Authors:** Veilumuthu Pattapulavar, Saranyadevi Subburaj, Sathiyabama Ramanujam, Priyadharshini Suresh Babu, Krishna Santhoshkumar, Anitha Kandasamy, Ramasamy Tamizhselvi, Divya Sharma, John Godwin Christopher

**Affiliations:** 1 Department of BioMedical Sciences, School of BioSciences and Technology, Vellore Institute of Technology (VIT), Vellore, Tamil Nadu, India; 2 Department of Biotechnology, School of BioSciences and Technology, Vellore Institute of Technology (VIT), Vellore, Tamil Nadu, India; 3 Department of Physics, School of Physics, Madurai Kamaraj University, Madurai, Tamil Nadu, India; 4 Department of Biosciences, School of Biosciences and Technology, Vellore Institute of Technology, Vellore, Tamil Nadu, India

**Keywords:** biosynthetic gene clusters, COX-2 (PTGS2), genome mining, *in silico* drug discovery, metabolomic profiling, natural product prioritization, putative anticancer metabolites, *streptomyces* sp. VITGV156 (MCC 4965)

## Abstract

**Introduction:**

*Streptomyces* species represent an important source of bioactive natural products, yet systematic genome-guided prioritization of metabolites targeting cyclooxygenase-2 (COX-2/PTGS2) remains limited. This study aimed to investigate the biosynthetic potential of Streptomyces sp. VITGV156 (MCC 4965) using an integrated genome mining and computational drug discovery pipeline.

**Methods:**

Whole-genome sequencing, functional annotation, antiSMASH v7.0.1-based biosynthetic gene cluster (BGC) prediction, LC-MS/MS metabolomic profiling, SwissADME analysis, target prediction, disease association mapping, molecular docking against PTGS2 (PDB: 5IKR), and PASS bioactivity prediction were performed to prioritize putative bioactive metabolites.

**Results:**

Genome analysis identified 29 predicted biosynthetic gene clusters, including clusters associated with geosmin, ectoine, albaflavenone, hopene, coelichelin, and SapB, together with several cryptic clusters exhibiting low similarity to known pathways. LC-MS/MS metabolomic profiling provided experimental support for active secondary metabolite production under the cultivation conditions employed. Computational prioritization identified PTGS2 (COX-2) as a biologically relevant target. Molecular docking demonstrated favorable binding affinities and interaction profiles for several predicted metabolites within the PTGS2 catalytic pocket. PASS analysis further suggested potential anticancer-related biological activities that require experimental validation.

**Discussion:**

These findings demonstrate the utility of integrating genome mining, metabolomic profiling, and computational drug discovery for prioritizing natural-product candidates. *Streptomyces* sp. VITGV156 (MCC 4965) represents a promising source of biosynthetic diversity and provides a genome-guided framework for identifying putative COX-2-targeting natural products for future experimental validation rather than confirming metabolite production or biological activity.

## Introduction

Metabolic reprogramming is now recognized as a defining hallmark of cancer, enabling malignant cells to sustain uncontrolled proliferation, survive hostile microenvironments, and evade immune surveillance. Dysregulated metabolic pathways not only fuel tumor growth but also shape inflammatory signalling, angiogenesis, and metastatic dissemination, thereby creating actionable vulnerabilities for therapeutic intervention (Zhao et al., 2021; Hindes et al., 2025). Among these interconnected pathways, inflammation-driven metabolic remodelling has emerged as a central driver of tumor initiation and progression, linking chronic inflammatory states to oncogenic transformation and cancer maintenance (Nishida and Andoh, 2025).

Cyclooxygenase-2 (COX-2), predicted by the PTGS2 gene, occupies a pivotal position at the intersection of metabolism, inflammation, and cancer. Unlike the constitutively expressed COX-1 isoform, COX-2 is inducible and frequently overexpressed in multiple malignancies, including hepatocellular carcinoma, breast cancer, colorectal cancer, and pancreatic cancer (Menter et al., 2010; Coutinho et al., 2024). COX-2–derived prostaglandins promote tumorigenesis by enhancing cell proliferation, suppressing apoptosis, stimulating angiogenesis, facilitating immune evasion, and remodelling the tumor microenvironment. Consequently, COX-2 has been widely investigated as a prognostic biomarker and therapeutic target in inflammation-driven cancers (Jin et al., 2023).

Despite the clinical success of nonsteroidal anti-inflammatory drugs (NSAIDs) and selective COX-2 inhibitors in reducing cancer risk and progression, their long-term use is constrained by cardiovascular, gastrointestinal, and renal toxicities (Lai et al., 2022; Ozleyen et al., 2022). These limitations underscore the urgent need for alternative COX-2–modulating agents with improved safety profiles, enhanced selectivity, and novel chemical scaffolds. Natural products continue to represent an unparalleled source of such scaffolds, owing to their structural complexity, biological relevance, and evolutionary optimization for molecular recognition (Hawkey and Langman, 2003; Zarghi and Arfaei, 2011; Derardja et al., 2024).

Microorganisms, particularly actinomycetes, have historically been the most productive contributors to clinically relevant natural products, including anticancer agents, immunomodulators, and enzyme inhibitors. Within this group, the genus *Streptomyces* is exceptional, encoding extensive biosynthetic repertoires that give rise to polyketides, non-ribosomal peptides, terpenoids, ribosomal synthesized and post-translationally modified peptides, and diverse hybrid metabolites (Alam et al., 2022; Newman and Cragg, 2026). Advances in whole-genome sequencing have revealed that the majority of *Streptomyces* biosynthetic gene clusters (BGCs) remain cryptic or poorly expressed under laboratory conditions, representing a vast reservoir of unexplored chemical diversity often referred to as microbial “biosynthetic dark matter” (Meesil et al., 2023).

The emergence of genome-guided natural product discovery has fundamentally transformed the field by enabling systematic exploration of this hidden metabolic potential. Bioinformatics platforms such as antiSMASH now allow comprehensive identification and annotation of BGCs, while integrative computational pipelines incorporating pharmacokinetic profiling, target prediction, disease association mapping, and structure-based modelling facilitate rational prioritization of metabolite–target pairs (Medema and Fischbach, 2015). This genome-to-drug paradigm offers a powerful alternative to traditional activity-guided isolation by directly linking genomic potential to disease-relevant molecular targets (Lee et al., 2025).

Recent evidence supports the feasibility of discovering COX-2–modulating compounds from microbial sources, including actinobacteria, validating microbial metabolism as a viable route for identifying inflammation-linked anticancer agents (Mohsin et al., 2022). However, systematic genome-driven strategies explicitly aimed at mapping microbial biosynthetic potential onto cancer-associated metabolic targets remain limited.

In this study, we apply an integrated genome-to-drug discovery framework to *Streptomyces* sp. VITGV156 (MCC 4965) is an endophytic strain isolated from the traditional tomato cultivar *Arka Rakshak* cultivated in Tamil Nadu, India. Through comprehensive genome mining, *in silico* drug-likeness and pharmacokinetic profiling, target prediction, disease association analysis, pathway validation, and structure-based molecular docking, we identify prostaglandin-endoperoxide synthase 2 (COX-2/PTGS2) as a convergent molecular target of multiple biosynthetically predicted metabolites. By linking microbial biosynthetic diversity to inflammation-driven oncogenic signalling, this work demonstrates a scalable strategy for translating microbial genomic potential into targeted metabolic therapies and highlights *Streptomyces* sp. VITGV156 as a promising source of COX-2–targeting natural products relevant to cancer intervention.

## Methods

### *Streptomyces* sp. VITGV156

A pure culture of *Streptomyces* sp. VITGV156 (MCC 4965) was characterized through both phenotypic and genotypic analyses (Veilumuthu and Christopher, 2022). Morphological assessment using phase-contrast and scanning electron microscopy provided detailed insights into its structural attributes. Additionally, a previously isolated strain, *Streptomyces* sp. VITGV156, stored at −20 °C in the Microbiology Lab at SBST, VIT University, was revived on ISP2 agar for further studies (Veilumuthu et al., 2024).

### Genome annotation and comparative genomic analysis

The whole genome of *Streptomyces* sp. VITGV156 was submitted to the NCBI SRA repository under Bio Project accession number PRJNA750872 (https://www.ncbi.nlm.nih.gov/bioproject/PRJNA750872); Bio Sample accession number SAMN20499087 (https://www.ncbi.nlm.nih.gov/biosample/20499087); SRA accession number SRS9645416 and Accession ID: 20499087 and NCMR (Culture Deposit Accession no)—MCC4965 (Pattapulavar et al., 2025). The datasets presented in this study can be found in online repositories. The names of the repository/repositories and accession number(s) can be found below: https://www.ncbi.nlm.nih.gov/biosample/20499087. The biosynthetic clusters involved in the synthesis of secondary metabolites were analyzed using antiSMASH v7.0.1 (Blin et al., 2023).

### Phylogenetic and phylogenomic analysis

The taxonomic position of strain VITGV156 was investigated using both 16S rRNA gene phylogeny and genome-based phylogenomic comparison, following current recommendations for *Streptomyces* systematics. The nearly complete 16S rRNA gene sequence was extracted from the assembled genome and compared with type strain sequences retrieved from the NCBI database. Multiple sequence alignment was performed using the ClustalW algorithm implemented in MEGA X. A phylogenetic tree was constructed using the Maximum Likelihood method with the Tamura–Nei model, and branch robustness was evaluated using 1,000 bootstrap replicates. To complement the 16S rRNA analysis, genome-scale relatedness was assessed using Average Nucleotide Identity (ANI) and digital DNA–DNA hybridization (dDDH) metrics derived from comparisons with closely related *Streptomyces* genomes identified through NCBI genome similarity searches. These metrics were interpreted using accepted species delineation thresholds (ANI ≈ 95–96%; dDDH ≈ 70%) (Veilumuthu et al., 2024).

### Metabolite production and extraction of secondary metabolites

To evaluate the biosynthetic potential of *Streptomyces* sp. VITGV156, the strain was cultivated in ISP2 broth supplemented with sugarcane bagasse and incubated at 30 °C for 15 days under aerobic conditions. For large-scale metabolite production, the organism was grown in 1,000 mL Erlenmeyer flasks containing 500 mL of ISP2 medium under identical cultivation conditions. Following incubation, the culture broth was centrifuged at 10,000 rpm for 20 min to separate the supernatant from the cellular biomass. Extracellular secondary metabolites were extracted from the culture supernatant using ethyl acetate (1:1, v/v). The mixture was vigorously mixed for 10 min and subsequently incubated on an orbital shaker at 200 rpm for 24 h to facilitate solvent partitioning. After phase separation, the organic layer was collected and concentrated using a rotary evaporator (RE100-Pro, DLAB Scientific, China) at 54 °C and 80 rpm. The resulting crude extract was dried, weighed, reconstituted in 200 μL methanol, and stored at −20 °C until further analysis. The extraction procedure was performed according to previously described methods with minor modifications (Nisha et al., 2025). To provide experimental support for genome-mining predictions, the crude ethyl acetate extract was subjected to GC–MS and LC–MS analyses for metabolite profiling and characterization of secondary metabolite diversity (Pattapulavar et al., 2025).

### LC–MS/MS analysis of the putative bioactive extract

Liquid chromatography–mass spectrometry (LC–MS/MS) analysis was performed to investigate non-volatile and higher-molecular-weight metabolites potentially associated with biosynthetic gene clusters predicted from the genome of *Streptomyces* sp. VITGV156 (Pattapulavar et al., 2026b). Chromatographic separation was carried out using a Phenomenex Kinetex C18 column (50 × 2.1 mm, 2.6 μm particle size, 100 Å pore size). Mass spectrometric detection was performed using a quadrupole time-of-flight (QTOF) mass spectrometer equipped with an electrospray ionization (ESI) source operating in negative ionization mode. The capillary voltage was maintained at 4.5 kV, the source temperature was set at 200 °C, and data were acquired at a scan rate of 1 Hz. Spectra were recorded over a broad m/z range to capture structurally diverse metabolite classes. Data-dependent MS/MS fragmentation experiments were performed on major precursor ions to obtain fragmentation patterns for metabolite annotation and comparison with the classes of natural products predicted through antiSMASH-based biosynthetic gene cluster analysis (Pattapulavar et al., 2026a).

### Predicting the target

A total of 29 secondary metabolite clusters from *Streptomyces* sp. VITGV156, predicted by antiSMASH v7.0.1 (Blin et al., 2023), was exposed to a post-screening study using the SwissADME server (Daina et al., 2017). Primarily, the canonical SMILES (Simplified Molecular Input Line Entry System) of the metabolites were retrieved from the PubChem repositories. Further, these SMILES were employed as an input in SwissADME to estimate the clusters based on their oral bioavailability and drug-likeness characteristics (Table 1). Following structural standardization, canonical SMILES were generated for all compound clusters and assigned unique identifiers. Each SMILES string was individually submitted to the SwissTargetPrediction tool using default settings, with *Homo sapiens* selected as the target organism (Gfeller et al., 2014). The retrieved output included predicted protein targets, target classes, UniProt IDs, and associated probability scores. Only targets with a probability ≥ 0.10 were kept in order to guarantee prediction reliability, creating a high-confidence target dataset for every cluster. After that, the datasets were combined to identify recurring or shared targets between the clusters. In order to link these targets to pertinent biological pathways and functional categories and provide a thorough grasp of their mechanistic roles, enrichment analyses were performed at the end.

**TABLE 1:** Genomic Features of *Streptomyces* sp. VITGV156.

Feature	Value
Genome assembly status	Draft genome
Total assembly size	7,207,566 bp
Number of scaffolds	201
N50 value	437,096 bp
G + C content	72.61%
Average nucleotide identity (ANI)*	91.22%
Digital DNA–DNA hybridization (dDDH)*	44.00%
Total predicted coding sequences (CDS)	6,386
Curated protein-coding genes	6,259
Hypothetical/uncharacterized CDS	127
rRNA operons	5
tRNA genes	81
BioSample ID	20499087
NCMR accession number	MCC 4965

*ANI and dDDH values were calculated against the closest phylogenomic neighbour, *Streptomyces luteus* TRM45540 (Luo et al., 2017).

### Target prediction by DisGeNET

Disease-gene correlations associated with the metabolite clusters were examined using the DisGeNET tool (Piñero et al., 2015). Gene symbols and UniProt identities were used to query the canonical gene targets and compound-associated proteins found in previous research. High-confidence results were prioritized based on DisGeNET scores and pertinent illness categories after the search yielded curated and literature-derived disease relationships. Comprehensive annotation, including related illness names, categories of evidence, source databases, and ontology IDs, was extracted for every target. A thorough illness association profile for the selected targets was then created by combining the obtained datasets, with a focus on finding common or recurring disease associations among several genes.

### Disease pathway validation

The Reactome repository, which combines in-depth computational evaluation with skilled manual curation, was used to further confirm the genome-predicted targets. High-resolution molecular annotations, such as enzyme activities, subcellular localization, and context-dependent conditions such as tissue and cell-type specificity, are provided by Reactome (Croft et al., 2011). Reliable functional interpretations of the annotated targets are ensured by supporting each pathway entry with experimental data from human research or deduced from evolutionarily conserved homologs in model organisms. Cyclooxygenase-2, which is predicted by the PTGS2 gene, was the most frequently found protein in our investigation. COX-2/PTGS2 acts as a pro-tumorigenic driver in liver cancer by stimulating inflammation, developmental signaling, angiogenesis, metastasis, and immune suppression. As a result, COX-2 is a crucial biomarker and treatment target for hepatocellular cancer. Hence, we investigate the function of COX-2/PTGS2 signaling in hepatocellular carcinoma and assess whether selective inhibition of PTGS2 expression can effectively suppress tumor cell proliferation, angiogenesis, and metastasis (Kassab, 2025).

### Binding affinity prediction

The three-dimensional crystal structure of COX-2 (PDB ID: 5IKR) was retrieved from the RCSB Protein Data Bank (Orlando et al., 2016). The protein structure was prepared by removing water molecules and heteroatoms, followed by the addition of polar hydrogen atoms and Kollman charges using AutoDock Tools. The co-crystallized ligand present in the structure was extracted and re-docked into the active site to validate the docking protocol. The accuracy of the docking method was assessed by calculating the root-mean-square deviation (RMSD) between the docked pose and the original crystallographic conformation, with an RMSD value of less than 2.0 Å considered acceptable. A known selective COX-2 inhibitor, Celecoxib, was used as a reference ligand to benchmark the docking results. Ligand structures of the selected clusters were energy-minimized and converted into the required PDBQT format. Docking simulations were performed using SeamDock, which integrates the AutoDock Vina scoring function (Murail et al., 2021). A grid box was defined to encompass the active site residues predicted from the co-crystallized ligand, with dimensions set to adequately cover the binding pocket (center coordinates: x = 2 Å, y = 7 Å, z = 1 Å; size: 57 × 48 × 52 Å). Docking parameters were maintained at default exhaustiveness levels unless otherwise specified. Multiple binding conformations were generated for each ligand, and the best pose was selected based on the lowest binding energy and favorable interaction profiles. Based on its binding energy, which suggests a stronger anticipated inhibitory relationship to COX-2, the top-ranked posture for each of the 13 clusters was selected. SeamDock generates different binding conformations using the AutoDock Vina scoring tool (Pattapulavar et al., 2026a). Key active-site residues, including Tyr130, Leu152, Pro153, and Lys468, were considered during interaction analysis, as they are known to play critical roles in ligand binding within the COX-2 active pocket.

### Interaction study

To understand the binding behavior of the ligands inside the COX-2 active site, an interaction study was performed in SeamDock following docking. By recognizing important non-covalent relations such as hydrogen bonds, hydrophobic contacts, π–π stacking, and electrostatic interactions created between the ligand and receptor residues, the platform offers comprehensive insights into molecular recognition. To ascertain how well each molecule fits into the catalytic pocket and remains stable within the binding environment, the 3D binding orientation and interaction maps were analyzed. In order to comprehend the possible inhibitory mechanism, the amino acid residues implicated in anchoring the chemicals were also examined. The selection of compounds with strong interaction patterns and stable binding was made possible by this methodical interaction assessment, which supports their potential as COX-2 inhibitors for the treatment of liver cancer.

### Antineoplastic and toxicity calculation

The predicted compounds were then analyzed using the PASS (Prediction of Activity Spectra for Substances) online program to identify potential pharmacological actions based on structural features (Lagunin et al., 2000). Two separate probabilities, Pa (probability of being active) and Pi (probability of being passive), each ranging from 0 to 1, are provided by PASS for each expected activity. Numerous biological characteristics are predicted by PASS. Generally speaking, a Pa value higher than 0.7 indicates that there is a good chance the molecule would exhibit the anticipated bioactivity under experimental conditions, making it worthy of additional investigation. Furthermore, the toxicity characteristics of the compounds were evaluated using the ProTox-II online platform, which provides a thorough assessment of their possible safety risks by estimating several toxicological endpoints, including hepatotoxicity, immunotoxicity, carcinogenicity, and mutagenicity (Banerjee et al., 2018).

## Results

### Genome analysis

**FIGURE 1:**
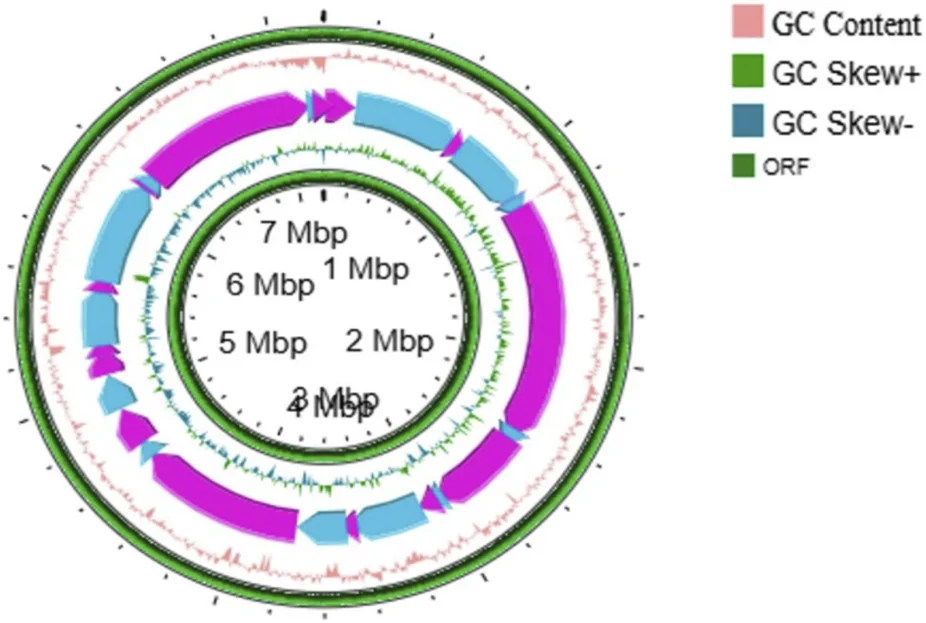
The structural organization of the *Streptomyces* sp. The VITGV156 genome was further examined through circular genome mapping (Figure 1). The map illustrates multiple concentric layers representing key genomic attributes, including coding sequences, GC content, and GC skew distribution.

Whole-genome sequencing of *Streptomyces* sp. VITGV156 revealed a linear chromosome with a total assembled length of 7,207,566 base pairs and a high G + C content of 72.61% (Figure 1). The genome assembly comprised 201 scaffolds, with an N50 value of 437,096 bp, indicating a reasonably contiguous draft genome. Annotation predicted 6,259 protein-coding genes, along with 5 rRNA operons and 81 tRNA genes. Comparative genomic analysis demonstrated that strain VITGV156 shares its closest phylogenomic relationship with *Streptomyces luteus* TRM45540, which was therefore selected as the reference strain for ANI and dDDH analyses. Notably, this strain was previously reported in genome databases under the designation *Streptomyces mutabilis* TRM45540 prior to its formal taxonomic classification as *Streptomyces luteus* sp. nov. The calculated Average Nucleotide Identity (ANI) value of 91.22% and digital DNA–DNA hybridization (dDDH) value of 44.00% were both below the accepted species delineation thresholds for bacterial species demarcation, indicating that strain VITGV156 likely represents a distinct and previously uncharacterized species within the genus *Streptomyces*. These findings strongly suggest that VITGV156 represents a previously uncharacterized *Streptomyces* species. The strain has been deposited in the National Centre for Microbial Resource (NCMR) under accession number MCC4965. Functional annotation based on Gene Ontology (GO) classification revealed diverse biological roles among the predicted coding sequences. Approximately 1,781 genes were associated with catalytic activity, molecular binding, and transport functions. In addition, 1,315 genes were linked to broader biological processes, while 851 genes were implicated in structural cellular components (Table 1). To explore its biosynthetic potential, the genome was analyzed using antiSMASH for the identification of biosynthetic gene clusters (BGCs). A total of 29 putative BGCs were detected, including clusters encoding non-ribosomal peptide synthetases (NRPS), ribosomally synthesized and post-translationally modified peptides (RiPPs), polyketides, terpenes, and siderophores. The metabolites listed in Table 2 represent biosynthetic products computationally predicted from antiSMASH-based BGC analysis and should not be interpreted as definitive evidence of metabolite production under the culture conditions used in this study. These predictions were used primarily to prioritize candidate natural products for downstream computational screening. Notably, three clusters exhibited less than 20% similarity to known biosynthetic pathways, indicating a strong potential for novel metabolite discovery. Among the predicted clusters, peptide-associated BGCs accounted for 34% (10 clusters), followed by polyketide synthase clusters at 24% (7 clusters), terpene clusters at 13% (4 clusters), and siderophore-related clusters at 10% (3 clusters). Additionally, metabolite profiling indicated the production of an indole-derived compound structurally distinct from previously reported analogs such as 5-isoprenylindole-3-carboxylate β-D-glycosyl ester, suggesting the presence of a potentially new chemical entity. Collectively, these genomic features highlight the significant biosynthetic diversity and untapped potential of *Streptomyces* sp. VITGV156 for novel secondary metabolite discovery (Table 2).

**TABLE 2:** Secondary metabolite biosynthesis-related gene cluster present in VITGV156, predicted using antiSMASH v7.0.1.

S. No.	Scaffolds	Types	Compounds	Similarity (in percentage)
1	Region 2.1	Indole	5-dimethylallylindole-3-acetonitrile	100
2	Region 3.2	T3PKS	Flaviolin/1,3,6,8-1,3 tetrahydroxy naphthalene	100
3	Region 7.1	Ectoine	Ectoine	100
4	Region 7.3	NI-siderophore	Desferrioxamine B	100
5	Region 12.1	Terpene	Albaflavenone	100
6	Region 17.2	Terpene	Geosmin	100
7	Region 18.2	Terpene	Hopene	100
8	Region 21.4	NRP- metallophoreNRPS	Coelichelin	100
9	Region 21.6	Lanthipeptide-class-III	Sap B, RiPP: Lanthipeptide	100
10	Region 3.1	NRPS-like	Streptothricin	95
11	Region 21.5	NRP-metallophore, NRPS, Lanthipeptide-class-I	Coelibactin	90
12	Region 18.4	T1PKS	Vicenistatin	70
13	Region 18.1	NRPS	CDA1/CDA2a	72
14	Region 13.1	T2PKS, PKS-like	Fluostatins M-Q	67
15	Region 12.2	T2PKS	Spore pigment	66
16	Region 2.2	Terpene	Isorenieratene	63
17	Region 11.1	Lanthipeptide-Class-III	Catenulipeptin	60
18	Region 21.3	RiPP-like	Informatipeptin	42
19	Region 20.1	T1PKS	Stambomycin	32
20	Region 18.3	Hydrogen-cyanide	Aborycin	28
21	Region 21.1	T1PKS	Streptovaricin	29
22	Region 16.1	NI-siderophore	Kinamycin	16
23	Region 19.1	T1PKS	Kendomycin B	15
24	Region 17.3	NI-siderophore	Paulomycin	11
25	Region 10.2	PKS-like, furan, lanthipeptide-class-v	Methylenomycin A	9
26	Region 15.1	Butyrolactone	Prejadomycin, Rabelomycin Gaudimycin C	6
27	Region 10.1	Polyketide: Type II polyketide	Granaticin	5
28	Region 21.2	Terpene	Versipelostatin	5
29	Region 7.2	Melanin	Istamycin	4

NRPS, Non-ribosomal peptide synthetase.

T1PKS, Type 1 PolyKetide Synthases.

T2PKS, Type 2 PolyKetide Synthases.

T3PKS, Type 3 PolyKetide Synthases.

RiPP, Ribosomally synthesized and post-translationally modified peptides.

### Circular genome visualization and structural features

The structural organization of the *Streptomyces* sp., the VITGV156 genome, was further examined through circular genome mapping (Figure 1). The map illustrates multiple concentric layers representing key genomic attributes, including coding sequences, GC content, and GC skew distribution. The outermost rings depict predicted open reading frames (ORFs) on both forward and reverse strands, highlighting the dense coding capacity typical of *Streptomyces* genomes. The inner rings represent GC content variation, showing minor fluctuations across the genome, consistent with the overall high GC percentage (72.61%). Additionally, the GC skew profile, represented by positive and negative deviations, provides insight into replication dynamics and potential origin/terminus regions. Notably, the genome exhibits a relatively balanced distribution of GC skew, suggesting a stable replication architecture without major asymmetry. Regions with pronounced GC skew shifts may correspond to replication origin sites or highly active transcriptional zones. The genome map also reflects the absence of large-scale structural rearrangements, supporting the integrity of the assembled genome. Overall, this circular representation provides a comprehensive overview of genome organization, reinforcing the high coding density and conserved structural features characteristic of actinomycetes.

### Phylogenetic analysis based on 16S rRNA gene

**FIGURE 2:**
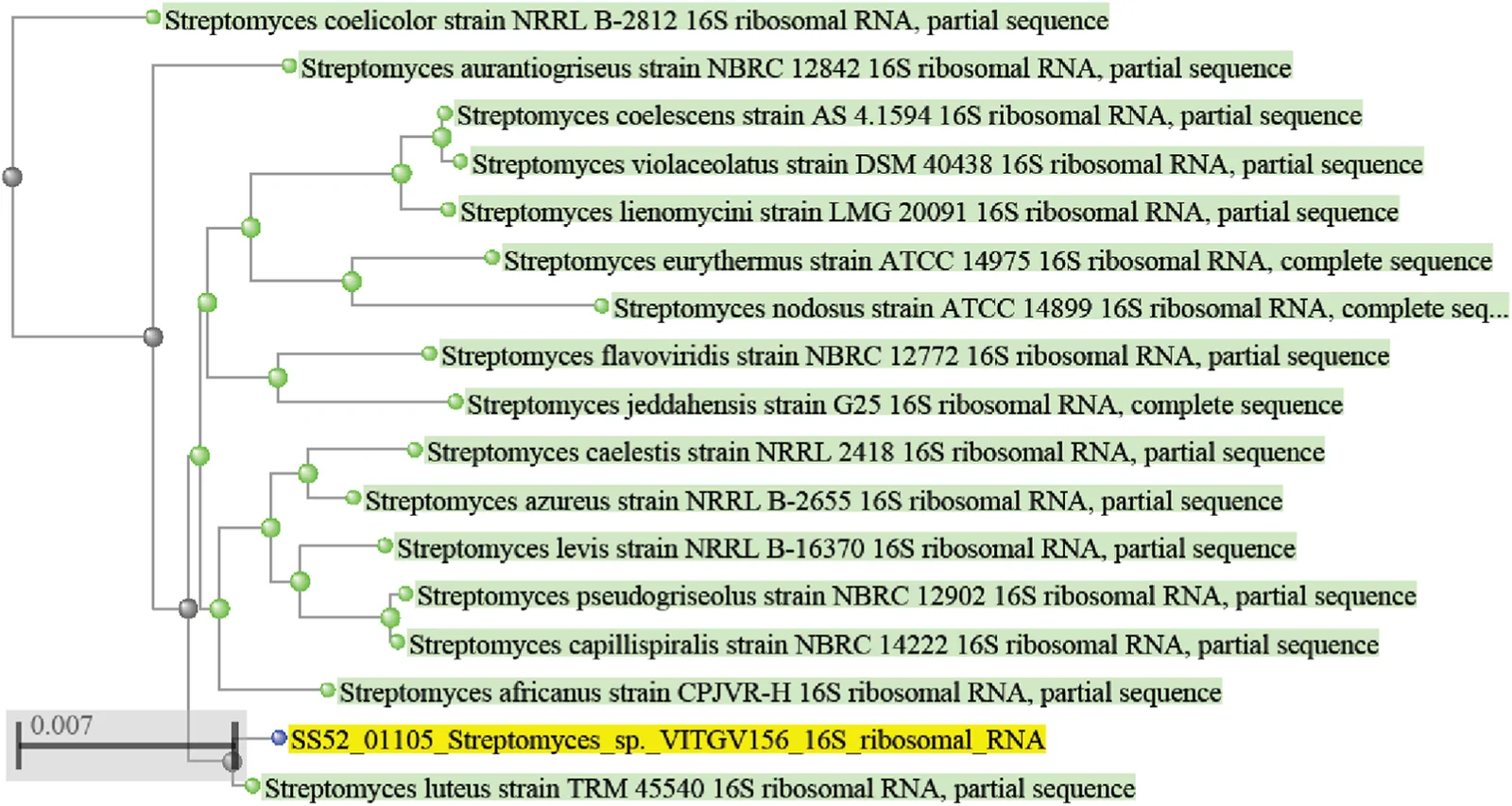
Phylogenetic tree based on 16S rRNA gene sequences showing the evolutionary position of *Streptomyces* sp. VITGV156 among closely related *Streptomyces* species. The tree illustrates the clustering of VITGV156 with *Streptomyces luteus* TRM45540 as its closest phylogenetic neighbour while maintaining a distinct lineage. Evolutionary distances are represented by branch lengths, and the scale bar indicates nucleotide substitutions per site.

To determine the evolutionary relationship of strain VITGV156, a phylogenetic tree was constructed using 16S rRNA gene sequences of closely related *Streptomyces* species (Figure 2). The analysis revealed that strain VITGV156 clusters within a well-supported clade comprising several *Streptomyces* taxa. Importantly, VITGV156 showed the closest phylogenetic proximity to *Streptomyces luteus* TRM45540, forming a distinct branch with a short evolutionary distance. Despite this close association, the strain remained clearly separated from its nearest phylogenetic neighbours, indicating notable genetic divergence. Other related species, including *S. coelicolor*, *S. violaceolatus*, and *S. azureus*, formed adjacent clusters but did not group directly with VITGV156, further supporting its distinct phylogenetic position. The branch lengths and bootstrap-supported nodes reinforced the robustness of the phylogenetic classification. Collectively, these findings, together with the ANI and dDDH values, provide strong evidence that *Streptomyces* sp. VITGV156 represents a potentially novel taxonomic entity within the genus *Streptomyces*.

### LC–MS/MS metabolomic evidence supporting genome-mined biosynthetic gene clusters

**FIGURE 3:**
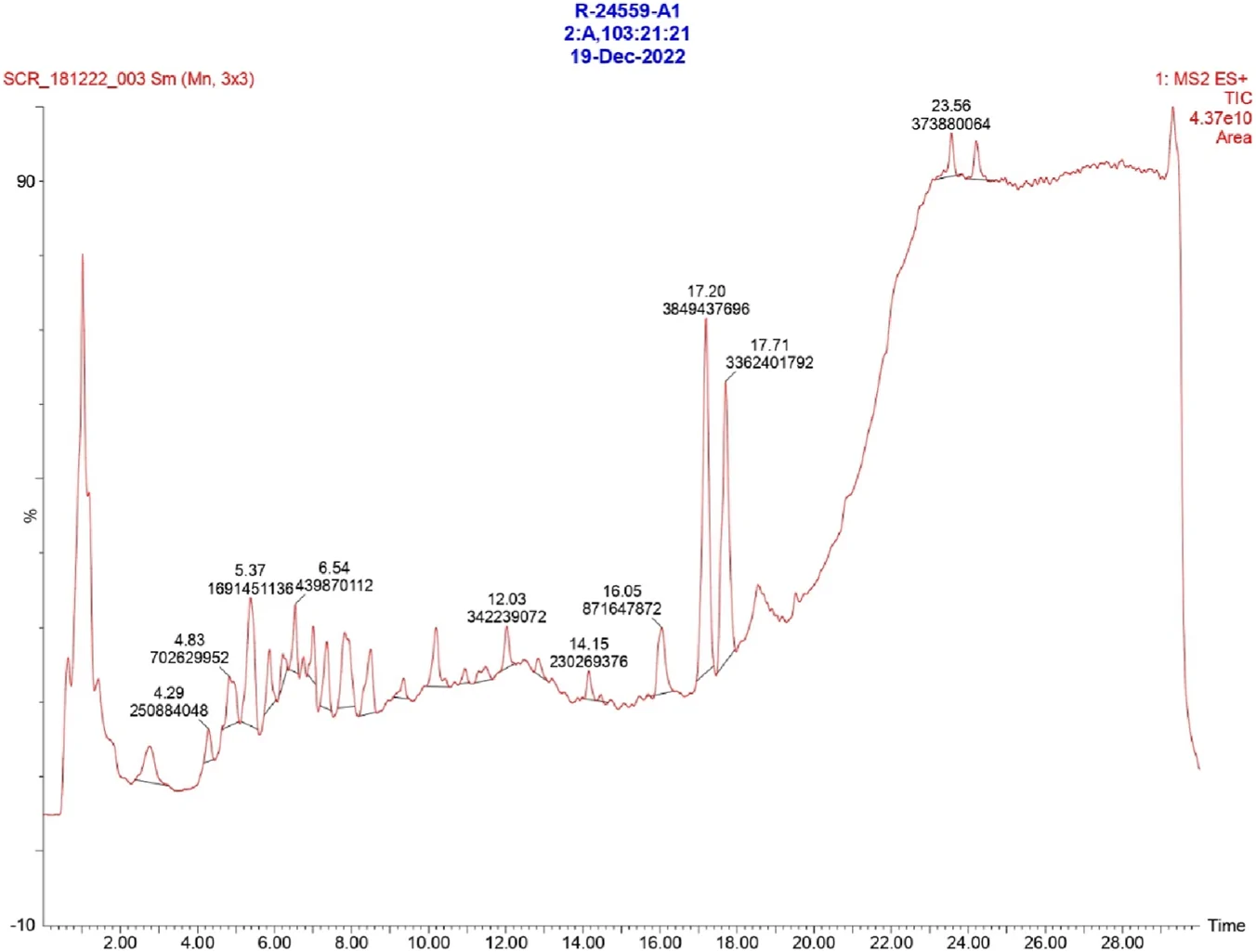
LC–MS base peak chromatogram (BPC) of the ethyl acetate extract of *Streptomyces* sp. VITGV156 acquired in negative electrospray ionization mode using QTOF-MS. The chromatogram exhibits numerous well-resolved peaks distributed across the chromatographic run, reflecting a chemically diverse secondary-metabolite profile. The predominance of signals between 10 and 27 min indicates the production of semi-polar and hydrophobic metabolites typical of actinomycetes. The observed metabolomic complexity provides experimental support for the multiple biosynthetic gene clusters predicted by genome mining.

To provide experimental support for the biosynthetic potential predicted through genome mining, the crude ethyl acetate extract of *Streptomyces* sp. VITGV156 was subjected to high-resolution LC–MS/MS analysis. The base peak chromatogram (Figure 3) revealed a chemically complex metabolite profile characterized by numerous well-resolved peaks distributed throughout the chromatographic run, indicating the production of diverse secondary metabolites under the cultivation conditions employed.

**FIGURE 4:**
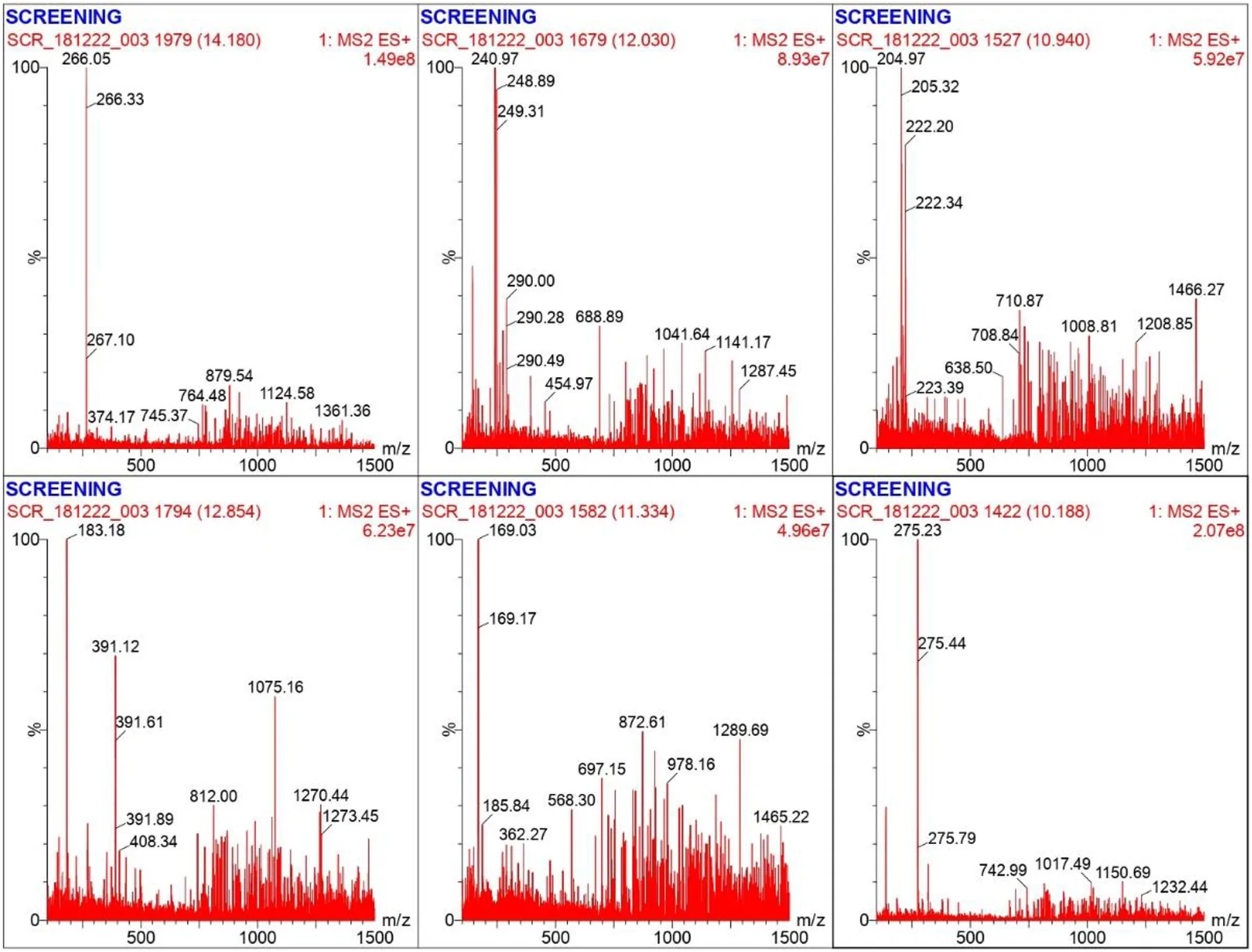
Representative LC–MS/MS fragmentation spectra of selected precursor ions detected from the ethyl acetate extract of *Streptomyces* sp. VITGV156. Major precursor ions ranging from m/z 169.03 to 1466.27 generated characteristic fragmentation patterns indicative of structurally diverse secondary metabolites. The coexistence of low-mass diagnostic fragments and high-molecular-weight precursor ions supports the presence of complex natural-product scaffolds potentially originating from terpene, polyketide, and peptide-associated biosynthetic pathways predicted by antiSMASH. Although definitive metabolite identification requires further structural characterization, these spectra provide metabolomic evidence supporting the active expression of multiple genome-predicted biosynthetic gene clusters.

Several prominent signals were observed between 10- and 27-min retention time, suggesting the presence of semi-polar and hydrophobic metabolites commonly associated with actinomycete-derived natural products. High-resolution mass spectrometric analysis detected precursor ions across a broad molecular range, extending from m/z 169.03 to m/z 1466.27. Representative precursor ions included m/z 169.03, 183.18, 204.97, 240.97, 275.23, 391.12, 568.30, 688.89, 697.15, 710.87, 812.00, 872.61, 978.16, 1008.81, 1041.64, 1075.16, 1141.17, 1208.85, 1232.44, 1270.44, 1287.45, 1289.69, 1465.22 and 1466.27. The detection of numerous ions within the m/z 350–1000 region together with several high-molecular-weight species exceeding m/z 1000 indicates substantial metabolite diversity and is consistent with the production of complex secondary metabolites (Figure 4).

Representative MS/MS spectra of selected precursor ions are shown in Figure 4. Fragmentation analysis generated multiple diagnostic product ions, demonstrating the presence of structurally diverse metabolite scaffolds. In particular, precursor ions detected at retention times 10.18, 10.94, 11.33, 12.03, and 12.85 min yielded characteristic fragmentation patterns suggestive of highly functionalized natural-product architectures. The occurrence of both low-mass diagnostic fragments and high-mass precursor ions supports the existence of metabolites derived from diverse biosynthetic pathways, including terpene-, polyketide-, and peptide-associated gene clusters predicted by antiSMASH.

Although definitive metabolite identification would require purification, NMR characterization, authentic standards, or molecular networking approaches, the LC–MS/MS data provide experimental metabolomic evidence that multiple biosynthetic pathways predicted within the genome are actively expressed under the tested culture conditions. These findings therefore complement the genome mining analysis and support the presence of a rich secondary-metabolite repertoire in *Streptomyces* sp. VITGV156.

### ADME studies

Altogether, 13 out of 29 compounds in the SwissADME investigation showed significant preliminary oral drug-likeness, with zero violations of Lipinski’s Rule of Five (Table 2). These beneficial physicochemical properties suggest that the chemicals have a high absorption potential. To determine their actual bioavailability, *in vitro* studies are still necessary. To progress these *in silico* prioritized in drug development pipelines, a thorough examination of numerous pharmacokinetic properties, such as solubility, membrane permeability, metabolic stability, and synthetic accessibility, is also essential. The ADME profile results for all 13 drugs are compiled in Table 3.

**TABLE 3:** ADME properties of the 13 compounds.

S. No.	Compounds	PubChem IDs	Canonical SMILES	Ro5 #violations
1.	5-dimethylallylindole-3-acetonitrile	129850907	CC(=CCC1=CC2=C(C=C1)NC=C2CC#N)C	0
2.	Flaviolin/1,3,6,8-1,3 tetrahydroxy naphthalene	160478	C1=C(C=C(C2=C1C(=O)C(=O)C=C2O)O)O	0
3.	Ectoine	126041	OC(=O)[C@@H]1CCN=C(N1)C	0
4.	Albaflavenone	25137938	O=C1C[C@@H]([C@@]23C1=C(C)C(C)(C)[C@H](C3)CC2)C	0
5.	Geosmin	29746	C[C@H]1CCC[C@@]2([C@]1(O)CCCC2)C	0
6.	Coelichelin	3247071	C[C@H]([C@H](C(=O)N(CCC[C@@H](C(=O)O)NC(=O)[C@@H](CCCN(C=O)O) N)O)NC(=O)[C@@H](CCCN(C=O)O)N)O	0
7.	Fluostatins M	139590584	C[C@H]1[C@H]([C@@H](C2=C3C(=C(C=C2C1=O)O)C4=C(C3=O)C=CC= C4OC)O)O	0
8.	Isorenieratene	9984420	C/C(=C\C=C\C=C(\C=C\C=C(\C=C\c1c(C)ccc(c1C)C)/C)/C)/C=C/C=C(/C=C/ c1c(C)ccc(c1C)C)\C	0
9.	Kendomycin B	145721314	C[C@H]\1CC[C@@H]2[C@@H]([C@@H]([C@H]([C@@H](O2)C3=C(C(=O)C= C4C3=C[C@@](O4)([C@H](C[C@@H](C/C(=C1)/C)C)C)O)O)C)C)C	0
10.	Methylenomycin A	10899285	OC(=O)[C@@H]1C(=C)C(=O)[C@@]2([C@@]1(C)O2)C	0
11.	Rabelomycin	443810	C[C@]1(CC2=CC(=C3C(=C2C(=O)C1)C(=O)C4=C(C3=O)C(=CC=C4)O)O)O	0
12.	Prejadomycin	50986198	CC1=CC(=O)[C@@H]2C3=C(C(=O)C[C@@]2(C1)O)C(=C4C(=C3)C=CC=C4O)O	0
13.	Istamycin	156086	CNC[C@@H]1CC[C@H]([C@H](O1)O[C@@H]2[C@H](C[C@@H]([C@H]([C@H] 2O)N(C)C(=O)CN)OC)N)N	0

### Target analysis

The SwissTargetPrediction website was used to discover the common predicted molecular targets among the 13 compounds (Table 4). These include matrix metalloproteinases (MMP1, MMP2, MMP3, MMP8, and MMP9), as well as PTGS2, and ALOX5. These proteins are associated with extracellular matrix degradation and inflammatory signalling pathways. Furthermore, metabolic regulation-related enzymes like 11-β-hydroxysteroid dehydrogenase 1 (HSD11B1), protein-tyrosine phosphatase 1B (PTPN1), and carbonic anhydrases (CA1, CA2, and CA4) were predicted as major targets, suggesting a possible impact on pH regulation and hormonal balance. Furthermore, PTGS2 is usually overexpressed in hepatocellular carcinoma, where it encourages tumor growth and persistent swelling. Its overexpression stimulates angiogenesis, cell proliferation, and resistance to apoptosis-mediated increased prostaglandin E2 signaling. Therefore, targeting PTGS2 may help reduce inflammation-driven tumor growth and enhance therapeutic results in liver cancer.

**TABLE 4:** Common target prediction for the 13 compounds using the SwissTargetPrediction server.

Uniprot ID	Protein name	Gene symbol
P28845	11-beta-hydroxysteroid dehydrogenase 1	HSD11B1
P35354	Cyclooxygenase-2	PTGS2
P14902	Indoleamine 2,3-dioxygenase	IDO1
P45452	Matrix metalloproteinase 13	MMP13
P14780	Matrix metalloproteinase 9	MMP9
P08253	Matrix metalloproteinase 2	MMP2
P22748	Carbonic anhydrase IV	CA4
P08254	Matrix metalloproteinase 3	MMP3
P22894	Matrix metalloproteinase 8	MMP8
P00918	Carbonic anhydrase II	CA2
P03956	Matrix metalloproteinase 1	MMP1
P22303	Acetylcholinesterase	ACHE
P18031	Protein-tyrosine phosphatase 1B	PTPN1
P00915	Carbonic anhydrase I	CA1
P29274	Adenosine A2a receptor (by homology)	ADORA2A
Q92731	Estrogen receptor beta	ESR2
P78536	ADAM17 (class - protease)	ADAM17
O00311	CDC7/DBF4 (cell division cycle 7-related protein kinase/Activator of S phase kinase)	CDC7
P0DMS8	Adenosine A3 receptor	ADORA3
P11309	Serine/threonine-protein kinase PIM1	PIM1
P04150	Glucocorticoid receptor	NR3C1
P03372	Estrogen receptor alpha	ESR1
P56817	Beta-secretase 1	BACE1
P09917	Arachidonate 5-lipoxygenase	ALOX5
P09874	Poly [ADP-ribose] polymerase-1	PARP1
P49354	Protein farnesyltransferase	FNTA

### DisGeNET analysis

Table 5 demonstrates the gene–disease relationships of the predicted targets along with their corresponding Reactome pathway IDs. Among the targets, PARP1 is highly associated with melanoma, whereas MMP9, a major matrix metalloproteinase involved in extracellular matrix breakdown, shows a high connection with liver cancer. Interestingly, PTGS2 frequently occurs with high clinical relevance scores, primarily linked to adenocarcinoma and liver carcinoma, suggesting a major role in inflammatory pathways that promote tumor growth. Furthermore, ESR1 is abundant in multiple signaling pathways linked to cancer and is linked to breast carcinoma. Overall, the recurring predominance of PTGS2 identifies it as a prominent pathogenic target for the screened drugs, especially in liver cancer regulation.

**TABLE 5:** Gene disease association for the predicted targets and their pathway IDs.

Gene symbol	Disease name	Score	Pathway ID
PARP1	Melanoma	1.0	R-HSA-162582, R-HSA-1643685, R-HSA-392499, R-HSA-73894, R-HSA-74160
MMP9	Liver carcinoma	1.0	R-HSA-1266738, R-HSA-1474244, R-HSA-162582, R-HSA-168256
PTGS2	Liver carcinoma	1.0	R-HSA-1430728, R-HSA-168256
PTGS2	Adenocarcinoma	1.0	R-HSA-1430728, R-HSA-168256
ESR1	Breast carcinoma	1.0	R-HSA-162582, R-HSA-1643685, R-HSA-392499, R-HSA-74160. R-HSA-8953897

Bolded genes PTGS2 were predicted as the most common disease with the 13 compounds.

### Pathway analysis

The PTGS2-associated pathway IDs, such as R-HSA-1430728 and R-HSA-168256, were submitted to the Reactome database for pathway validation to verify the involvement of genes in pertinent biological signaling networks. Particularly, inflammation-driven metabolic changes in tumor cells are facilitated by PTGS2-associated pathways, such as R-HSA-1430728. Liver cancer modifies glycogen turnover to promote ongoing proliferation while increasing glycolysis and lactate production to maintain rapid ATP synthesis. Increased fatty acid synthesis, cholesterol ester formation, and eicosanoid signaling processes in which PTGS2 plays a critical regulatory role in driving inflammation, angiogenesis, and tumor progression are other notable changes in lipid metabolism. Hepatocellular carcinoma also renovates metabolic networks to promote malignancy and prevent cell death, as seen by increased amino acid usage, nucleotide production, and mitochondrial malfunction (Li et al., 2015).

Moreover, the reactome-based immune system pathway analysis shows the involvement of the PTGS2-associated route (R-HSA-168256) in immune regulation. By regulating prostaglandin-mediated immune activation and cytokine production, PTGS2 plays a crucial part in inflammatory signaling. In the innate immune system, macrophages, neutrophils, dendritic cells, and NK cells respond to pathogens through Toll-like receptor (TLR) activation, leading to downstream cytokine signaling. These innate responses initiate adaptive immunity, where antigen-presenting cells (APCs) activate CD4^+^ and CD8^+^ T cells, resulting in clonal expansion, cytotoxic responses against infected or malignant cells, and B-cell development into plasma cells generating antibodies. Dysregulation of PTGS2-driven inflammatory pathways increases chronic inflammation, immune evasion, and tumor growth, which are hallmarks of liver cancer (Duan et al., 2022). Therefore, PTGS2 is linked to important immunological mechanisms that support carcinogenic transformation and tumor immune escape.

### Docking and interaction studies

**FIGURE 5:**
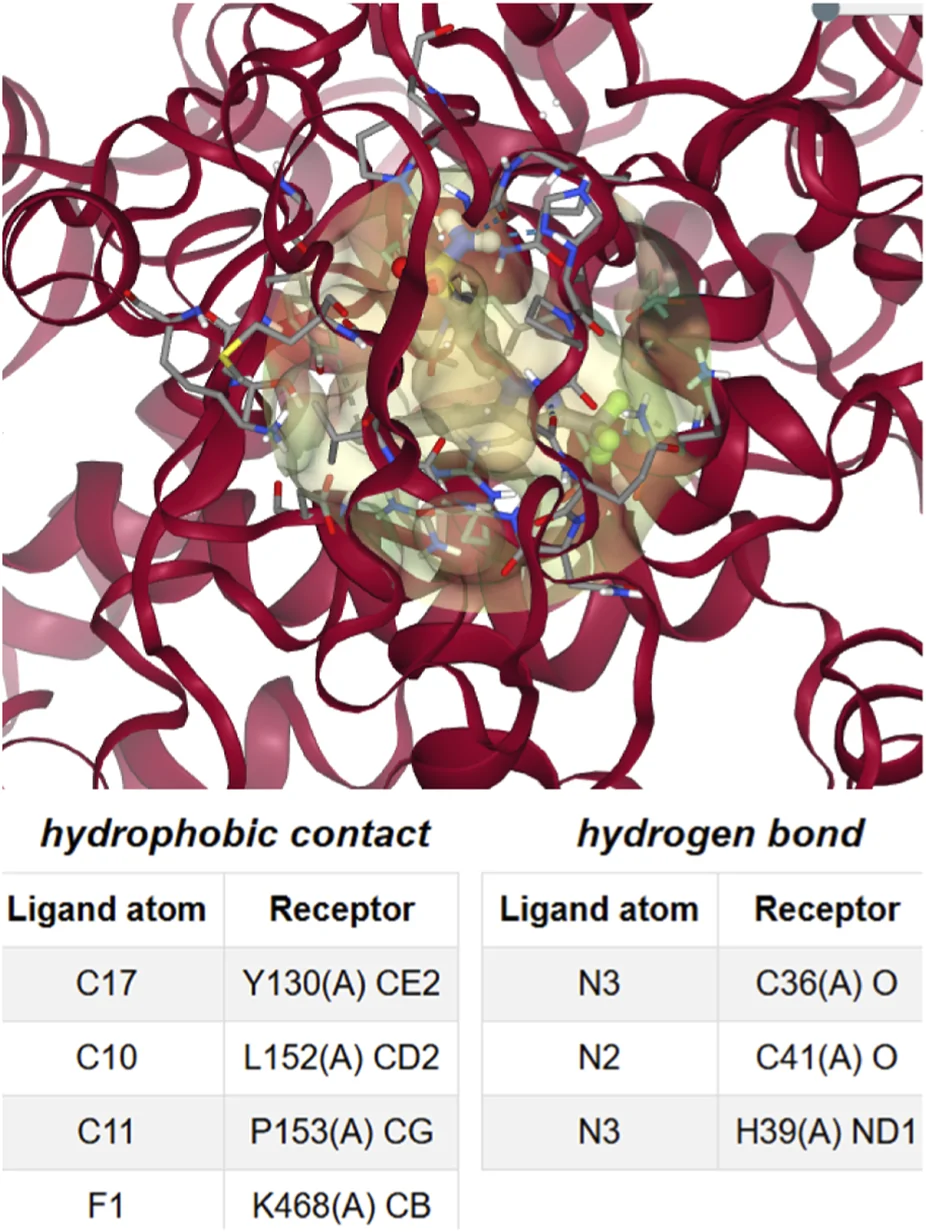
Binding pocket and its catalytic sites of 5IKR with Celecoxib (Reference). Molecular docking of celecoxib within the catalytic pocket of COX-2 (PDB ID: 5IKR). The co-crystallized inhibitor occupies the canonical active site and establishes interactions with key catalytic residues responsible for substrate recognition and inhibition. Re-docking of celecoxib produced an RMSD value of 1.25 Å, validating the docking protocol and providing a reference framework for evaluating the binding behaviour of the predicted microbial metabolites.

The molecular docking study of 13 putative bioactive compounds and reference compound celecoxib against the COX-2 protein is displayed in Table 6, with binding affinities ranging from −5.7 to −10.4 kcal/mol. In our study, docking score was treated as a critical parameter for assessing binding potential. Compounds with lower binding affinities were not considered strong candidates despite favourable pharmacokinetic properties. With the higher negative binding energy of −10.4 kcal/mol, rabelomycin showed the highest interaction among these, followed by isorenieratene (−10 kcal/mol), prejadomycin (−9.9 kcal/mol), and kendomycin B (−9.4 kcal/mol), all of which showed a high potential to bind and inhibit COX-2. Moreover, these 3 compounds demonstrated higher binding affinity toward COX-2 than the reference inhibitor Celecoxib (−9.3 kcal/mol), as evidenced by their more negative binding energy values, suggesting a potentially stronger inhibitory interaction (Figure 5). Fluostatins M also demonstrated a substantial binding score (−9.2 kcal/mol), but drugs like ectoine and istamycin A compounds demonstrated considerably lesser affinities. Overall, the *in silico* prioritized compounds with larger negative binding energies, rabelomycin, isorenieratene, and prejadomycin suggest potential binding affinity and stronger interaction within the active site of COX-2, indicating them as attractive compounds for further *in silico* prioritize optimization and *in vitro* validation against inflammation-associated liver cancer. Moreover, the docking protocol was validated by re-docking the co-crystallized ligand (Celecoxib) into the COX-2 active site, yielding an RMSD value of 1.25 Å, which falls within the acceptable range (<2.0 Å), thereby confirming the accuracy of the docking methodology. However, these findings require further validation through molecular dynamics simulations and experimental assays, which will be incorporated in future studies. The interaction pattern observed for rabelomycin closely overlaps with residues involved in celecoxib recognition, suggesting a plausible binding mode within the PTGS2 catalytic pocket. The docking orientation of prejadomycin indicates stabilization through hydrophobic interactions within the substrate-binding channel.

**TABLE 6:** Docking scores of 13 compounds against the target COX-2.

S. No.	Name of the compounds	PubChem IDs	Binding affinity (kcal/mol)
PDB ID: 5IKR			
1.	Celecoxib (Reference)	2662	−9.3
2.	5-dimethylallylindole-3-acetonitrile	129850907	−7.8
3.	Flaviolin/1,3,6,8-1,3 tetrahydroxy naphthalene	160478	−7.3
4.	Ectoine	126041	−5.7
5.	Albaflavenone	25137938	−7.3
6.	Geosmin	29746	−6.8
7.	Coelichelin	3247071	−6.3
8.	Fluostatins M	139590584	−9.2
9.	**Isorenieratene**	**9984420**	**−10**
10.	Kendomycin B	145721314	−9.4
11.	Methylenomycin A	10899285	−6.6
12.	**Rabelomycin**	**443810**	**−10.4**
13.	**Prejadomycin**	**50986198**	**−9.9**
14.	Istamycin A	156086	−6.2

Bolded compounds demonstrated better binding affinity.

**FIGURE 6:**
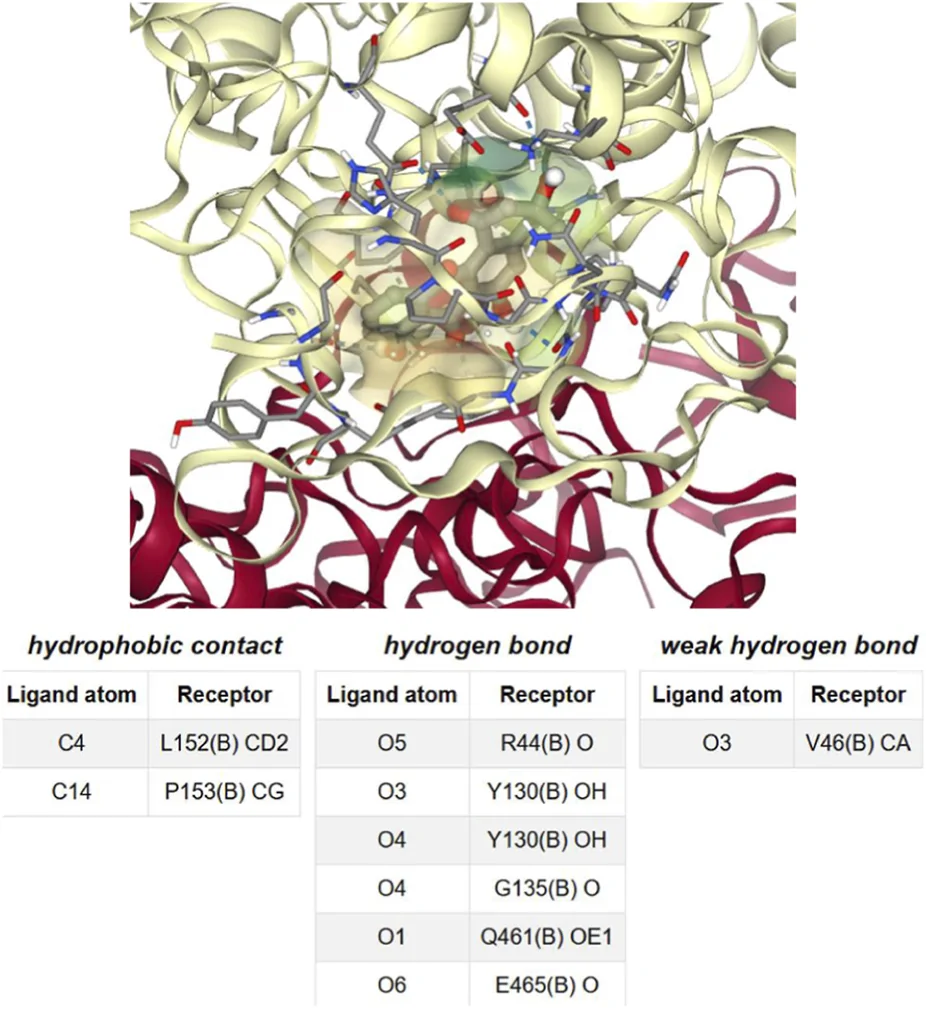
Binding pocket and its catalytic sites of 5IKR with Rabelomycin. Binding mode of rabelomycin within the COX-2 catalytic pocket (PDB ID: 5IKR). Rabelomycin exhibited the strongest predicted binding affinity among all screened compounds (−10.4 kcal/mol), forming multiple hydrogen-bond interactions with residues R44, Y130, G135, Q461, and E465, together with hydrophobic contacts involving L152 and P153. These interactions contribute to stable accommodation within the catalytic region and support its prioritization as a putative COX-2-targeting candidate for future validation.

Rabelomycin generates multiple strong hydrogen bonds with key residues such as R44, Y130, G135, Q461, and E465 when it binds to the catalytic region of the 5IKR receptor. The stability and binding selectivity of the receptor are enhanced by these connections. Its location within the active site is further improved by hydrophobic interactions with L152 and P153. Overall, the interaction profile indicates that Rabelomycin shows strong affinity for 5IKR, suggesting potential binding to the catalytic site (Figure 6). However, this functional inhibition requires future experimental validation.

**FIGURE 7:**
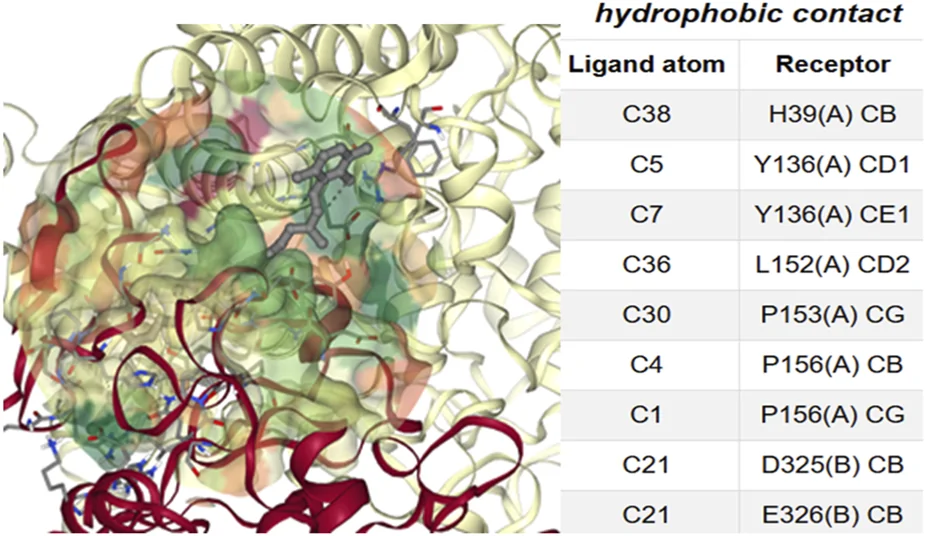
Binding pocket and its catalytic sites of 5IKR with Isorenieratene. Binding orientation of isorenieratene within the COX-2 active site (PDB ID: 5IKR). Isorenieratene displayed a favorable docking score (−10.0 kcal/mol) and was predominantly stabilized through extensive hydrophobic interactions with residues H39, Y136, L152, P153, P156, D325, and E326. The interaction pattern suggests efficient occupation of the hydrophobic channel of COX-2 and highlights the potential of carotenoid-like metabolites as scaffolds for anti-inflammatory drug discovery.

Isoenieratene is efficiently accommodated by the hydrophobic core of the 5IKR binding pocket, which is mainly stabilized by a number of nonpolar interactions with significant residues like H39, Y136, L152, P153, P156, D325, and E326. These significant hydrophobic contacts, which also show a strong ligand–receptor fit, suggest that isorenieratene may successfully occupy and clog the catalytic site, thereby affecting 5IKR function (Figure 7).

**FIGURE 8:**
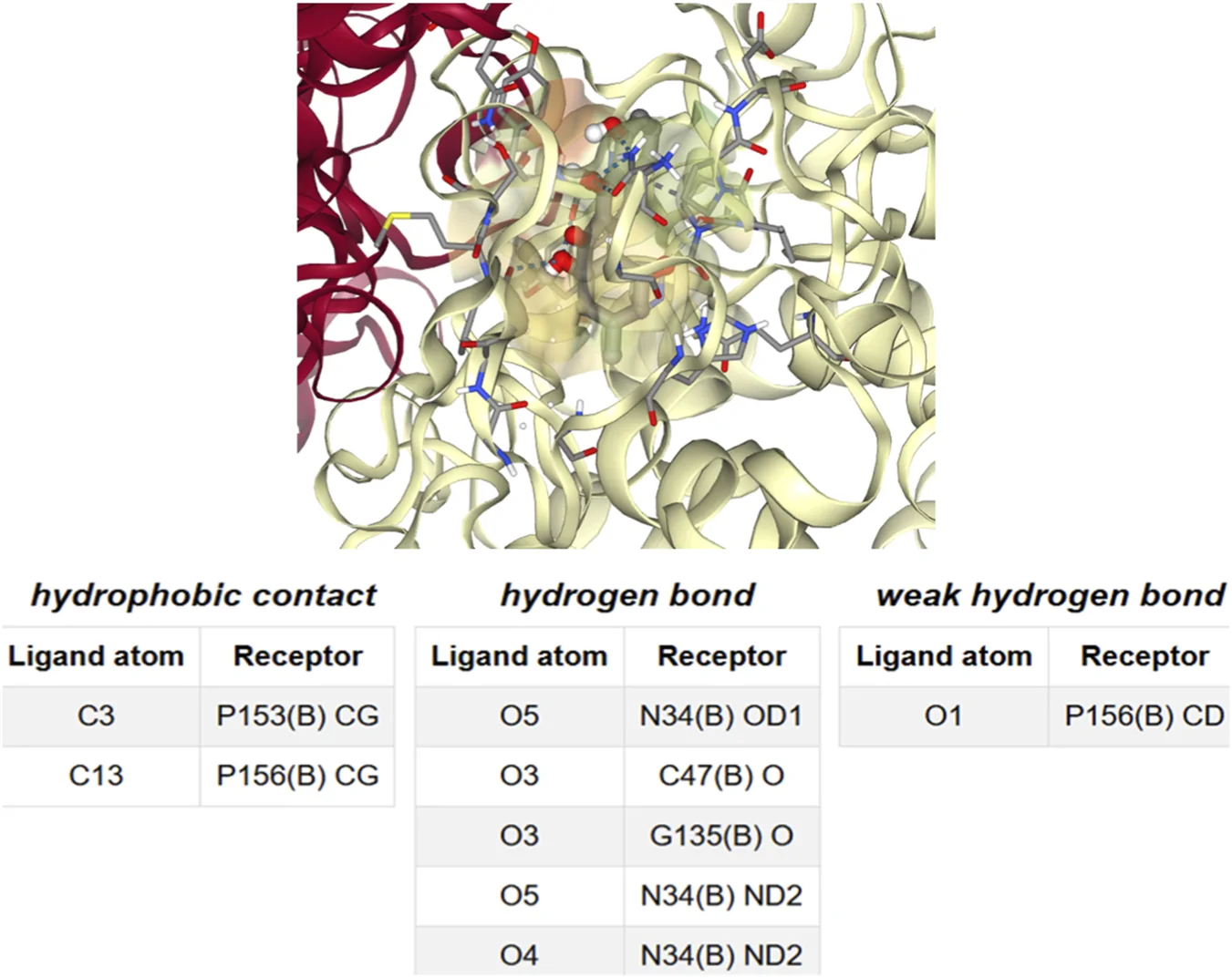
Binding pocket and catalytic sites of 5IKR with Prejadomycin. Molecular docking of prejadomycin with COX-2 (PDB ID: 5IKR). Prejadomycin demonstrated a strong predicted binding affinity (−9.9 kcal/mol) and occupied the catalytic cavity through a combination of hydrogen-bonding and hydrophobic interactions. Although fewer interactions were observed compared with rabelomycin, the compound maintained stable positioning within the active site, supporting its selection as one of the top-ranked *in silico* prioritized candidates.

The hydrophobic core of the 5IKR binding pocket accommodates isorenieratene effectively, and it is primarily stabilized by several nonpolar interactions with important residues such as H39, Y136, L152, P153, P156, D325, and E326. Isorenieratene may successfully occupy and obstruct the catalytic site, potentially impairing 5IKR function, according to these substantial hydrophobic interactions, which also support a robust ligand–receptor fit. Multiple hydrogen bonds and hydrophobic interactions within the catalytic region contributed to Rabelomycin’s greatest binding affinity toward 5IKR among the three ligands. Isorenieratene ranked second, displaying substantial hydrophobic stabilization, while Prejadomycin showed comparatively fewer interactions and the lowest binding score (Figure 8).

### Anticancer and toxicity analysis

With high Pa values for overall antineoplastic activity, the PASS prediction results show that all three *in silico* prioritized compounds have significant anticancer potential. Rabelomycin’s vast therapeutic importance is suggested by its wide range of projected effectiveness over a variety of tumor types, particularly non-Hodgkin lymphoma, breast, lung, bladder, brain, and liver cancers. Isoenieratene shows significant antineoplastic potential in addition to other hepatoprotective and hepatic-related therapeutic activities, with particularly high prognoses for lymphoma, brain, breast, and lung cancers. Even though it is still active, prejadomycin has much lower Pa values and a narrow range of predicted cancer targets, most notably lung, pancreatic, breast, and ovarian cancers. The prediction profile indicates that Rabelomycin is the most effective anticancer contender overall. Prejadomycin and isorenieratene come next, suggesting that they might be tested further as multi-target antineoplastic medications (Table 7). The PASS prediction outcomes for the three *in silico* prioritized compounds confirmed the substantial antineoplastic potential with various cancer-type-specific effects. Significantly, Rabelomycin, Isorenieratene, and Prejadomycin all showed the anticipated effect on liver cancer, indicating their significance for the management of hepatocellular carcinoma. The most extensive and predicted anticancer profile was shown by Rabelomycin, which was followed by Isorenieratene and Prejadomycin.

**TABLE 7:** Anticancer activity of the 3 *in silico* prioritized compounds.

P_a_	P_i_	Activity
Rabelomycin		
0.861	0.006	Antineoplastic
0.497	0.003	Antineoplastic antibiotic
0.491	0.040	Antineoplastic (non-Hodgkin’s lymphoma)
0.364	0.039	Antineoplastic (breast cancer)
0.337	0.015	Antineoplastic (small cell lung cancer)
0.284	0.040	Antineoplastic alkaloid
0.254	0.013	Antineoplastic (bladder cancer)
0.258	0.023	Antineoplastic (endocrine cancer)
0.270	0.041	Antineoplastic (brain cancer)
0.252	0.034	Antineoplastic (lymphoma)
0.235	0.028	Antineoplastic (glioblastoma multiforme)
0.240	0.054	Antineoplastic (lung cancer)
0.194	0.026	Antineoplastic (gastric cancer)
0.276	0.108	Antineoplastic (multiple myeloma)
0.177	0.017	Antineoplastic (uterine cancer)
0.208	0.049	Antineoplastic (sarcoma)
0.194	0.039	Antineoplastic (ovarian cancer)
0.186	0.042	Antineoplastic (glioma)
0.206	0.078	Antineoplastic (liver cancer)
0.158	0.032	Antineoplastic, alkylator
0.158	0.033	Antineoplastic (squamous cell carcinoma)
0.203	0.090	Antineoplastic enhancer
0.177	0.070	Antineoplastic (melanoma)
0.189	0.094	Antineoplastic (non-small cell lung cancer)
0.162	0.087	Antineoplastic (renal cancer)
0.228	0.168	Antineoplastic (solid tumors)
0.215	0.159	Antineoplastic (pancreatic cancer)
0.115	0.067	Antineoplastic (carcinoma)
Isorenieratene		
0.881	0.005	Antineoplastic
0.610	0.013	Antineoplastic (non-Hodgkin’s lymphoma)
0.392	0.009	Antineoplastic (brain cancer)
0.408	0.035	Antineoplastic (solid tumors)
0.313	0.051	Antineoplastic (breast cancer)
0.246	0.010	Hepatocyte nuclear factor 4 alpha antagonist
0.246	0.010	Hepatocyte nuclear factor antagonist
0.239	0.025	Antineoplastic (glioblastoma multiforme)
0.218	0.015	Antineoplastic antibiotic
0.214	0.103	Hepatic disorders treatment
0.242	0.043	Antineoplastic (non-small cell lung cancer)
0.224	0.026	Antineoplastic (bladder cancer)
0.205	0.039	Antineoplastic (cervical cancer)
0.238	0.108	Antineoplastic (small cell lung cancer)
0.179	0.156	Hepatoprotectant
0.174	0.047	Antineoplastic (ovarian cancer)
0.178	0.062	Antineoplastic (renal cancer)
0.186	0.076	Antineoplastic (lung cancer)
0.169	0.062	Antineoplastic (glioma)
0.158	0.090	Antineoplastic (melanoma)
0.135	0.129	Antineoplastic (lymphoma)
0.084	0.079	Heparan-alpha-glucosaminide N-acetyltransferase inhibitor
Prejadomycin		
0.771	0.015	Antineoplastic
0.347	0.032	Antineoplastic (lung cancer)
0.281	0.045	Antineoplastic (small cell lung cancer)
0.238	0.013	Antineoplastic antibiotic
0.203	0.029	Antineoplastic (thyroid cancer)
0.256	0.099	Antineoplastic (pancreatic cancer)
0.218	0.085	Antineoplastic (breast cancer)
0.170	0.049	Antineoplastic (ovarian cancer)
0.200	0.094	Antineoplastic alkaloid
0.186	0.082	Antineoplastic (glioblastoma multiforme)
0.169	0.077	Antineoplastic (melanoma)
0.304	0.219	Antineoplastic (non-Hodgkin’s lymphoma)
0.185	0.115	Antineoplastic enhancer
0.161	0.108	Antineoplastic (endocrine cancer)
0.144	0.102	Antineoplastic (glioma)
0.157	0.116	Antineoplastic (bladder cancer)
0.159	0.143	Antineoplastic (liver cancer)
0.134	0.129	Antineoplastic (lymphoma)

According to the ProTox-II investigation, none of the three substances was harmful in terms of cyto-, immuno-, or hepato-toxicity. Rabelomycin is significantly more hazardous than the other two, as seen by its lowest LD_50_, indicating considerably higher toxicity than the other two. Overall, Isorenieratene is the safest, followed by Prejadomycin, whereas Rabelomycin requires more toxicity assessment (Table 8).

**TABLE 8:** Toxicity analysis by the ProTox-II algorithm.

Toxicity	Isorenieratene	Rabelomycin	Prejadomycin
Hepatotoxicity	Non-toxic	Non-toxic	Non-toxic
Cytotoxicity	Non-toxic	Non-toxic	Non-toxic
Immunotoxicity	Non-toxic	Non-toxic	Non-toxic
Predicted toxicity class	5	4	4

## Discussion

The present study employed a genome-guided, *in silico* genome-predicted framework to prioritize putative bioactive secondary metabolites predicted by *Streptomyces* sp. VITGV156 (MCC 4965) and to map their potential relevance to inflammation-associated cancer pathways. By integrating biosynthetic gene cluster mining with pharmacokinetic filtering, target prediction, disease association analysis, pathway validation, and structure-based molecular docking, we systematically reduced chemical and biological complexity to identify prostaglandin-endoperoxide synthase 2 (PTGS2/COX-2) as a convergent and biologically plausible molecular target.

It is important to note that the metabolites listed in Table 2 were predicted through antiSMASH-based biosynthetic gene cluster prediction and therefore represent the genomic biosynthetic potential of *Streptomyces* sp. VITGV156 rather than experimentally purified or structurally validated metabolites. Consequently, the present study should be interpreted as a genome-guided computational prioritization framework rather than definitive experimental confirmation of metabolite production or biological activity. The detection of metabolite features spanning a broad molecular-weight range, including high-mass ions characteristic of complex secondary metabolites, provides preliminary metabolomic support for the biosynthetic diversity predicted through antiSMASH analysis. To partially address the gap between genomic prediction and metabolite production, LC–MS/MS profiling of the crude ethyl acetate extract was performed. The resulting chromatographic and mass spectral data demonstrated the presence of numerous secondary metabolites spanning a broad molecular mass range (m/z 169–1466), consistent with the metabolic complexity predicted by antiSMASH analysis. While these data do not allow definitive assignment of individual metabolites to specific biosynthetic gene clusters, they provide experimental evidence that multiple biosynthetic pathways are transcriptionally and metabolically active under the cultivation conditions employed. Consequently, the present study integrates genomic prediction with preliminary metabolomic validation, thereby strengthening confidence in the biosynthetic potential of *Streptomyces* sp. VITGV156 while acknowledging that targeted metabolite isolation and structural characterization remain necessary for definitive confirmation. Not all predicted metabolites were expected to function as direct COX-2 inhibitors. Certain compounds, including ectoine and geosmin, were retained in the analysis because they are widely recognized *Streptomyces*-associated metabolites with reported biological relevance in stress adaptation, antimicrobial activity, anti-inflammatory responses, and cancer-associated pathways. For example, ectoine has been reported to exhibit cytoprotective, anti-inflammatory, antioxidant, and immunomodulatory properties, while geosmin and related terpenoid metabolites have previously demonstrated antimicrobial and anticancer-associated activities in genome-guided metabolite screening studies. These observations support their inclusion as biologically relevant metabolites within the broader computational screening pipeline, even though their direct COX-2 inhibitory activity remains experimentally unverified.

Future investigations involving metabolite isolation, purification, expression analysis of biosynthetic gene clusters, quantitative metabolomics, and biochemical COX-2 inhibition assays will be necessary to establish direct links between biosynthetic pathways, metabolite production, and biological function. Nevertheless, genome mining coupled with computational prioritization has emerged as an effective preliminary strategy for accelerating natural-product discovery from *Streptomyces* species, particularly for identifying cryptic or low-expression biosynthetic pathways with potential pharmaceutical relevance.

Initial ADME profiling using SwissADME revealed that 13 out of 29 predicted metabolites compounds demonstrated that favorable physicochemical properties with no violations of Lipinski’s Rule of Five, indicating acceptable preliminary oral drug-likeness. While these parameters do not confirm bioavailability, they serve as an essential early-stage filter to prioritize compounds for downstream evaluation and reduce attrition in later drug development stages (Daina et al., 2017). Importantly, structurally diverse metabolites spanning polyketides, terpenoids, siderophores, and aminoglycoside-like scaffolds passed these criteria, underscoring the broad pharmacological potential embedded within the *Streptomyces* biosynthetic repertoire.

Target prediction analysis revealed a heterogeneous but biologically coherent set of predicted protein targets, including matrix metalloproteinases (MMPs), carbonic anhydrases, metabolic enzymes, nuclear hormone receptors, and inflammatory mediators. Among these, PTGS2 emerged as a recurring target across multiple compounds, supported by high prediction confidence. COX-2 plays a central role in linking metabolic remodeling to inflammatory signaling in cancer, primarily through prostaglandin E2–mediated effects on angiogenesis, immune suppression, tumor cell proliferation, and resistance to apoptosis (Greenhough et al., 2009; Wang and Dubois, 2010). The convergence of multiple chemically distinct metabolites on PTGS2 strengthens the biological relevance of this target within the screened metabolite set and supports its prioritization for further analysis.

Disease association mapping using DisGeNET further reinforced the pathogenic relevance of PTGS2, revealing strong associations with liver carcinoma and adenocarcinoma. Hepatocellular carcinoma is characterized by chronic inflammation, altered lipid metabolism, and dysregulated eicosanoid signaling, processes in which COX-2 is known to function as a pro-tumorigenic driver (Li et al., 2015). The repeated identification of PTGS2 in disease-linked pathways, alongside MMP9 and ESR1, suggests that the predicted metabolites may influence tumor progression through interconnected inflammatory, metabolic, and extracellular matrix remodeling mechanisms rather than via a single isolated pathway.

Reactome-based pathway analysis contextualized PTGS2 within broader metabolic and immune signaling networks relevant to liver cancer. PTGS2-associated pathways were linked to lipid metabolism, prostaglandin synthesis, inflammatory cascades, and immune regulation. These findings are consistent with established models of cancer metabolic reprogramming, wherein inflammatory enzymes such as COX-2 reshape the tumor microenvironment by modulating immune cell recruitment, cytokine production, and angiogenic signalling (Pavlova and Thompson, 2016; Duan et al., 2022). Importantly, our analysis does not imply direct metabolic inhibition but rather highlights PTGS2 as a metabolic-inflammatory nexus that may be modulated by microbial natural products.

Structure-based molecular docking provided additional support for PTGS2 as a feasible binding target. Several compounds, particularly rabelomycin, isorenieratene, and prejadomycin, exhibited favorable binding energies within the COX-2 catalytic pocket. Rabelomycin demonstrated the strongest predicted affinity, stabilized by multiple hydrogen bonds and hydrophobic interactions with key active-site residues, suggesting a stable and specific ligand–protein interaction. While docking scores alone cannot predict inhibitory efficacy, they provide a mechanistic rationale for prioritizing these compounds for experimental validation (Murail et al., 2021). Notably, the structural diversity of the top-ranking ligands suggests that multiple chemotypes may engage COX-2 through distinct interaction modes, potentially enabling selective modulation.

PASS-based activity prediction further supported the anticancer relevance of the prioritized compounds, particularly rabelomycin and isorenieratene, which showed high probabilities for antineoplastic activity across multiple cancer types, including liver cancer. These predictions are hypothesis-generating and should be interpreted cautiously; however, their concordance with target prediction and docking results strengthens confidence in the biological plausibility of the predicted *in silico* prioritized compounds (Lagunin et al., 2000). Toxicity assessment using ProTox-II suggested acceptable safety profiles for all three *in silico* prioritized compounds, with isorenieratene displaying the most favorable predicted toxicity parameters. Rabelomycin, while showing the strongest binding affinity, exhibited higher predicted toxicity, emphasizing the importance of balancing potency with safety during *in silico* prioritized optimization. (Lagunin et al., 2000).

Collectively, this study demonstrates the utility of a genome-guided, computational genome-predicted compounds for translating microbial biosynthetic potential into disease-relevant molecular hypotheses. Rather than claiming therapeutic efficacy, our findings prioritize *Streptomyces*-derived metabolites as candidate COX-2 modulators and establish a rational framework for subsequent experimental validation. Future work should focus on biochemical COX-2 inhibition assays, cellular inflammation and cancer models, and structure–activity relationship studies to confirm and refine the predicted interactions. By integrating microbial genomics with systems-level pharmacology, this approach contributes to the expanding toolkit for metabolism-oriented cancer drug discovery. From a broader perspective, the integration of genome mining, metabolomic profiling, and computational drug discovery contributes to sustainable natural-product research and aligns with global efforts to promote human health, innovation-driven biotechnology, and responsible utilization of microbial resources Importantly, the work aligns with Sustainable Development Goals (SDGs)—notably SDG 3 (Good Health and Wellbeing) through anticancer drug discovery, SDG 9 (Industry, Innovation and Infrastructure) via advanced bioinformatics-driven pipelines, and SDG 12 (Responsible Consumption and Production) by promoting sustainable bioprospecting.

The present investigation primarily represents a genome-guided computational prioritization framework. Although preliminary LC–MS/MS metabolomic profiling provided evidence of active secondary-metabolite production, definitive structural identification of individual metabolites was not performed. Furthermore, direct associations between specific biosynthetic gene clusters and detected metabolites remain to be experimentally established. Likewise, the predicted interactions between prioritized metabolites and PTGS2 were derived from computational analyses and have not yet been validated through biochemical, cellular, or *in vivo* studies. Consequently, the findings should be interpreted as hypothesis-generating observations that provide a foundation for future experimental investigation.

## Conclusion

This study highlights the value of integrating genome mining, metabolomic profiling, bioinformatics, and computational pharmacology to explore the biosynthetic potential of *Streptomyces* sp. VITGV156 (MCC 4965). Genome analysis revealed a diverse repertoire of biosynthetic gene clusters, including several cryptic and underexplored pathways, indicating a substantial capacity for secondary metabolite biosynthesis. AntiSMASH-based predictions identified multiple putatively associated metabolites, which were subsequently subjected to pharmacokinetic screening, target prediction, molecular docking, and activity prediction analyses. The integration of these computational approaches enabled the prioritization of several putative COX-2 (PTGS2)-targeting metabolites, with rabelomycin, isorenieratene, and prejadomycin emerging as the most promising *in silico* candidates based on their predicted interaction profiles and binding affinities. Importantly, preliminary LC–MS/MS metabolomic profiling provided experimental evidence supporting active secondary-metabolite production and partially validated the biosynthetic potential inferred from genome mining. Nevertheless, the detected metabolites were not definitively identified, and direct links between specific biosynthetic gene clusters and individual metabolites remain to be established. Taken together, this study presents a genome-guided and computational framework for the prioritization of natural-product candidates from microbial resources. While the findings provide valuable insights into the biosynthetic landscape of *Streptomyces* sp. VITGV156 and identify promising candidates for future investigation, metabolite isolation, structural characterization, biochemical COX-2 inhibition assays, cell-based studies, and *in vivo* validation will be required to confirm their production, biological activity, and therapeutic relevance. From a broader perspective, the integration of genome mining, metabolomic profiling, and computational drug discovery contributes to sustainable natural-product research and supports ongoing efforts toward health innovation, biotechnology development, and responsible utilization of microbial resources.

## Data Availability

The whole genome of *Streptomyces* sp. VITGV156 was submitted to the NCBI SRA repository under BioProject accession number PRJNA750872 (https://www.ncbi.nlm.nih.gov/bioproject/PRJNA750872), BioSample accession number SAMN20499087 (https://www.ncbi.nlm.nih.gov/biosample/20499087), SRA accession number SRS9645416 and Accession ID: 20499087 and NCMR (Culture Deposit Accession no)—MCC4965.
